# Anaplastic Lymphoma Kinase (ALK)-Positive Large B-cell Lymphoma in Children: A Case Report and Review of Literature

**DOI:** 10.7759/cureus.102952

**Published:** 2026-02-04

**Authors:** Ryan Dananjaya, Agnes S Harahap, Teny T Sari, Ganda Ilmana, Novie A Chozie, Amanda V Ardhiawan, Maria F Ham

**Affiliations:** 1 Anatomical Pathology Department, Dr. Cipto Mangunkusumo/Universitas Indonesia, Jakarta, IDN; 2 Medical Education and Research Institute, Human Cancer Research Center, Jakarta, IDN; 3 Hematology Oncology Division, Child Health Department, Dr. Cipto Mangunkusumo/Universitas Indonesia, Jakarta, IDN; 4 Faculty of Medicine, Universitas Indonesia, Jakarta, IDN

**Keywords:** anaplastic lymphoma kinase, cancer chemotherapy, case report, large b-cell lymphoma, pediatrics and oncology

## Abstract

Anaplastic lymphoma kinase-positive large B-cell lymphoma (ALK⁺-LBCL) is an aggressive and rare B-cell lymphoma caused by the ALK gene mutation. It is exceptionally rare in children and typically presents at advanced stages. Due to morphological mimicry of other hematologic malignancies and conditions, diagnosis remains challenging. Moreover, prognosis is poor as there is a lack of standard therapy. We report the case of a 13-year-old Indonesian male who presented with abdominal pain, initially presumed to be appendicitis. Multiple abdominal masses were subsequently identified following surgery. Histopathological examination revealed diffuse sheets of large round cells with plasmablastic morphology and numerous mitotic figures. Immunohistochemical analysis demonstrated positive ALK expression with a granular cytoplasmic pattern, weak CD45 expression, strong positivity for CD38, MUM1, and EMA, a Ki-67 proliferation index of approximately 70%, and negativity for B- and T-cell markers. Staging confirmed ALK⁺-LBCL, stage IV, according to the Murphy and St. Jude Children’s Research Hospital staging system, with both nodal and extranodal involvement. The patient was treated with CHOP chemotherapy, with alectinib added from the second cycle. A partial response was achieved after four cycles and was sustained for more than one year. Notably, neuron-specific enolase levels increased in parallel with disease progression. This case highlights diagnostic challenges related to an unusual clinical presentation and pathological overlap with other hematologic diseases.

## Introduction

Non-Hodgkin lymphoma (NHL) is the most common hematological malignancy worldwide, comprised of a diverse and heterogeneous group of cancers arising from the lymphoid tissues [1]. Accounting for 2.8% of all malignancies in 2022, this group occurs predominantly in Asian and European countries [2]. NHL has heterogeneity in mutation, morphology, and behavior [3]. In rare cases, anaplastic lymphoma kinase (ALK) gene mutation could be found [4]. ALK-positive large B-cell lymphoma (ALK^+^-LBCL), according to the World Health Organization (WHO), is an aggressive B-cell lymphoma with an expression of fusion protein and plasmablastic/immunoblastic morphology [5]. Almost 60% of patients admitted to hospitals are diagnosed at advanced stages (III/IV) [2,4,6]. ALK^+^-LBCL has a prevalence of less than one percent among the total LBCL, with a male predominance reported in adults [5]. ALK^+^-LBCL is especially rare in pediatrics, as only 20 cases have been reported around the globe, resulting in limited data on pediatric presentation and unclear gender distribution [5,7].

Nodal involvement is the most common symptom of ALK^+^-LBCL, accompanied by B-symptoms such as weight loss, fever, and night sweats in more than half of the patients [5]. Besides its rarity, diagnosing ALK^+^-LBCL may be challenging due to both morphological and immunophenotypes resembling other hematolymphoid and non-hematolymphoid neoplasms. Morphologically, this type exhibits monomorphic cells with prominent central nucleoli that resemble immunoblastic cells, with abundant cytoplasm [4,8-10]. Giant Hodgkin/Reed-Sternberg-like cells were also reported, which can lead to a misdiagnosis of classic Hodgkin lymphoma [4,8-10].

Due to its rarity, no standardized therapy for this lymphoma has been established. The National Comprehensive Cancer Network (NCCN) suggested clinical trials for treatment options. Together with an aggressive clinical behavior, high relapse rate, and poor response to initial therapy such as cyclophosphamide, doxorubicin, vincristine, and prednisone (CHOP) or CHOP-derived regimens, the five-year survival is only as low as 34% [8,11]. Here, we report a rare case involving a 13-year-old patient who initially underwent surgery for suspected appendicitis, but was subsequently diagnosed with ALK^+^-LBCL, confirmed through histopathological examination and extensive immunohistochemical analysis.

## Case presentation

**Figure 1:**
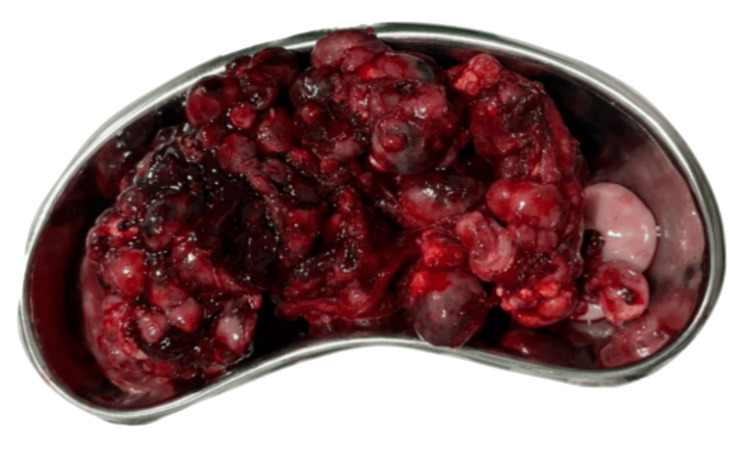
Multiple intra-abdominal round fragile masses of variable sizes.

A 13-year-old Indonesian male patient presented with a chief complaint of intermittent abdominal pain over the past two months, accompanied by bloating, without any visible or palpable mass observed. Slight weight loss was noted. One month later, the patient complained of unusual abdominal pain in the right lower quadrant (RLQ), suspected to be acute abdomen due to appendicitis. No history of chronic illness, immunodeficiency disorder, immunosuppressive agent use, or malignancy was noted. The patient was already suggested to undergo surgery, but refused due to improved conditions with pain-killer medications. Two weeks after the initial visit, the patient was sent to the hospital with worsened pain in the RLQ, accompanied by fever. An appendicogram examination confirmed appendicitis, and the patient and family agreed to undergo surgery. During the surgery, multiple round masses of variable sizes were found, and the masses were partially resected due to the difficulty of the procedure (Figure 1). The patient was referred to Cipto Mangunkusumo Hospital for advanced diagnosis and treatment.

**Figure 2:**
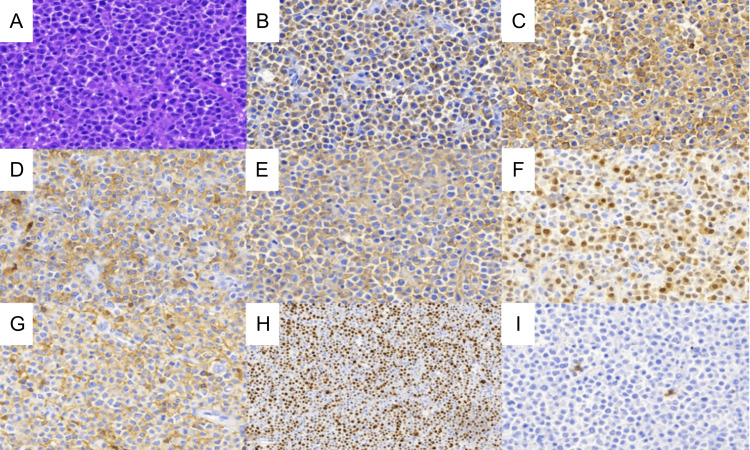
(A) Histopathology appearance of the tumor showed diffuse large and round tumor cells (hematoxylin and eosin staining). Immunohistochemistry examination showed positive for (B) ALK (granular pattern in the cytoplasm). (C) CD138. (D) CD10. (E) EMA. (F) MUM1. (G) CD45. (H) Ki-67 Positive in more than 70% of tumor cells, and negative for (I) CD30.

Morphologically, this case shows diffuse large cells with plasmablastic features (Figure 2). The immunophenotype showed positivity in ALK, CD10, CD38, CD45, CD138, multiple myeloma oncogene 1 (MUM1), epithelial membrane antigen (EMA), and 70% Ki-67 proliferation index. Tumor was negative for T cell markers (CD1a, CD3, CD4, CD8, and CD43), natural killer (NK) markers (CD56), histiocyte markers (CD68), lymphoid precursors (terminal deoxynucleotidyl transferase (TdT)), progenitor cells (CD34, CD117), and other B cell markers (CD20, CD79a, and paired box protein 5 (PAX5)) (Table 1). At this time, no other visible or palpable masses were observed. Chemotherapy was recommended, but the family refused.

**Table 1:** Complete immunophenotyping results.

Marker	Results
CD45	Positive (weak expression)
CD38, MUM1, EMA	Positive
ALK	Positive (granular cytoplasmic)
Ki67	Positive (70%)
CD1a, CD3, CD4, CD8, CD43, CD20, CD79a, PAX5, CD56, CD68, TdT, CD34, CD117, MPO, S100, SALL4, HMB45, INI1, myoD1, chromogranin, synaptophysin, CD99, NSE, calretinin, INSM, Phox2B	Negative
Kappa; lambda	Inconclusive

**Figure 3:**
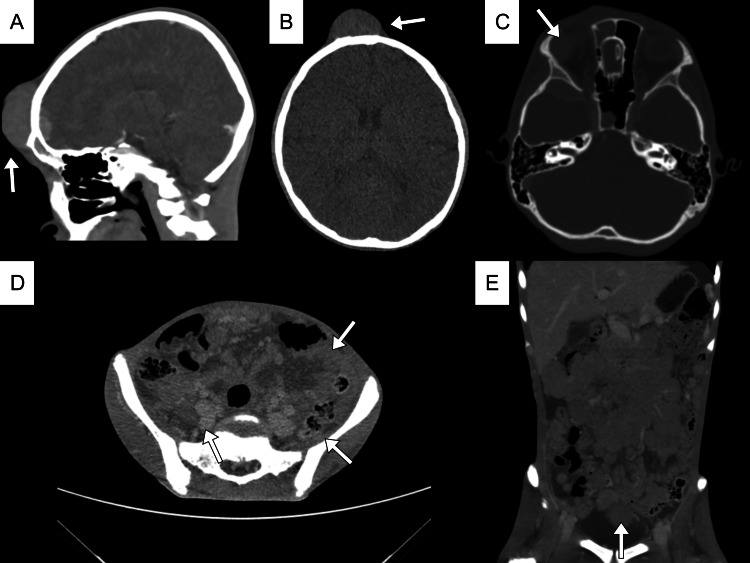
Brain and abdominal CT scan. (A-C) Brain CT scan shows multiple masses at the frontal bone, also found at the right intraorbital side of the eye. (D-E) Abdominal CT scan showed conglomerating masses at the paraaortic and mesentery areas extending to the pelvic region.

Two months later, the patient returned with visible, enlarging masses at the forehead, trunk, and inguinal area, along with severe cancer pain and moderate malnutrition. The patient also reported visual disturbances, raising suspicions of a retroorbital mass, which prompted further scans (Figure 3). Multiple solid lesions with malignant characteristics at the right frontal bone, temporal, and intraorbital areas, compressing the parenchymal lobes and right ocular bulb, were noted in the brain scan. An abdominal scan showed multiple lymphadenopathies conglomerating around the paraaortic and mesentery areas, extending from the abdomen to the pelvic area, suggestive of lymphoma. These masses narrowed the left renal vein and inferior vena cava, and no pathologic bone lesions were found. Laboratory findings showed high thrombocytosis (823 × 109/L), slight anemia (10.4 g/dL), lymphopenia (11.8%), and mild hyponatremia (130 mEq/L) with no correction needed. Along with signs of progressive disease, neuron-specific enolase (NSE) was increased from 50.3 ng/mL to 110 ng/mL (normally <5 ng/mL). Lactate dehydrogenase (LDH) was high (694 U/L, normal range is 125-220 U/L). The bone marrow aspiration result was positive for tumor cells, while the cerebrospinal fluid (CSF) was negative.

**Figure 4:**
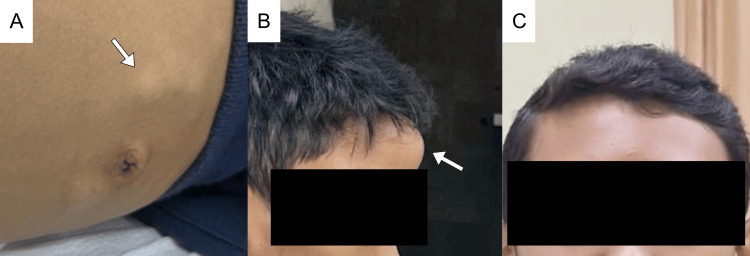
Clinical presentation progression of the patient. (A) Pre-chemotherapy multiple lymphadenopathies in the abdomen and (B) a solid mass at the frontal bone. (C) Improvement of patient condition and decrease of frontal bone mass post-chemotherapy.

The patient was diagnosed as ALK+-LBCL, stage IV (Murphy and St. Jude Children’s Research Hospital Staging System), and high-risk Group B French-American-British/Lymphome Malin B (FAB/LMB). Chemotherapy with the CHOP regimen was started. The pain improved significantly, and the mass visibly reduced in size. A second-generation ALK inhibitor, alectinib, was given as adjuvant therapy. Patient tolerated the treatment well, with moderate nausea, vomiting, and slight anemia and neutropenia observed during the chemotherapy period. Levels of LDH were monitored routinely and were always within the normal range. No palpable mass was observed clinically, and he achieved normal nutritional status (Figure 4).

**Figure 5:**
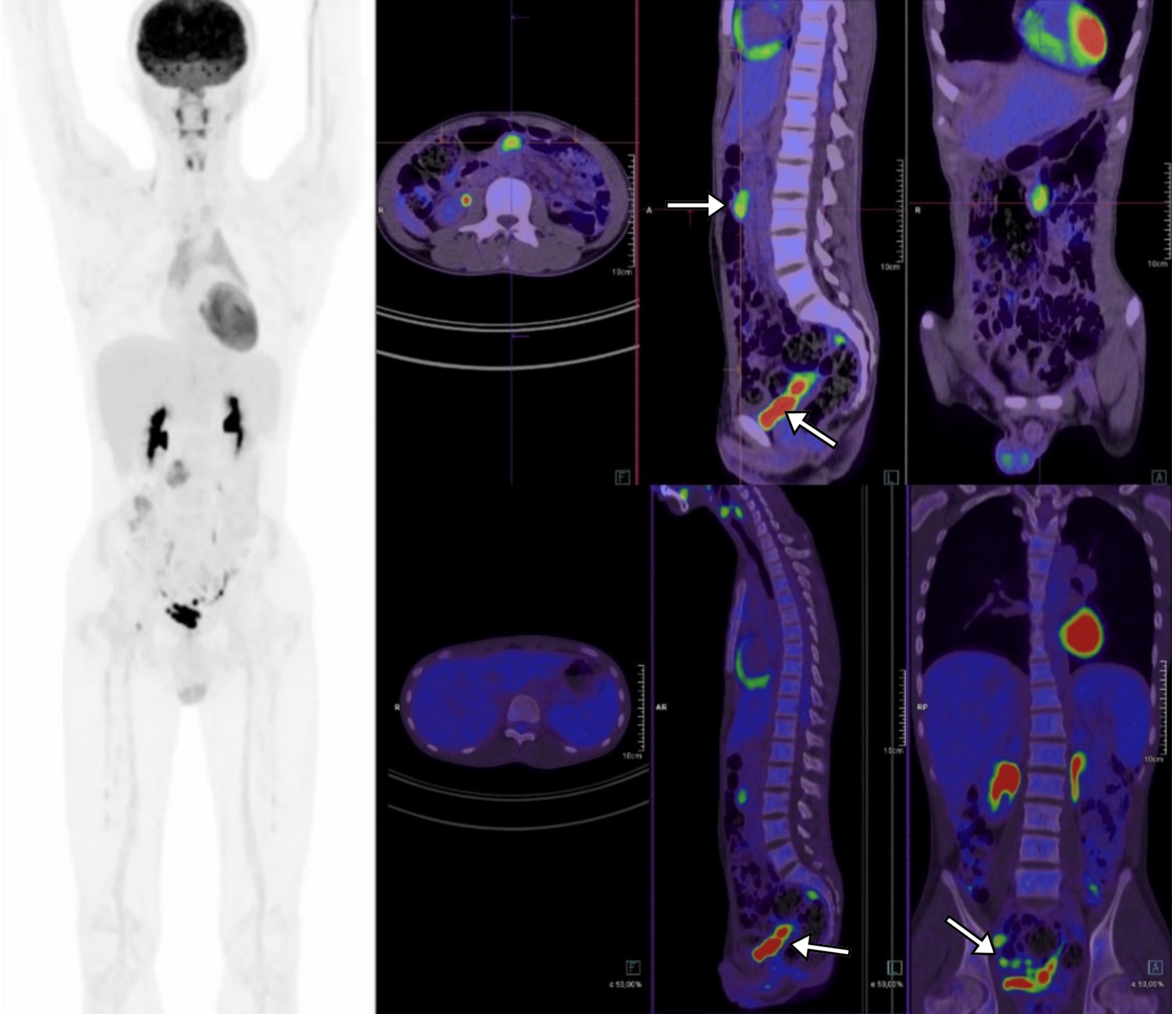
FDG PET/CT follow-up scan after the fourth cycle of chemotherapy showed residual lymphadenopathies (white arrows) in the mesentery, pelvis, and left and right para-aortic areas. No residual masses were found at the right frontal, temporal, intraorbital, and costal regions. Additionally, no involvement of the liver, bones, bone marrow, or other organs was found.

After four cycles of CHOP, 18F-fluorodeoxyglucose (FDG) positron emission tomography/computed tomography (PET/CT) was performed. Significantly reduced sizes of lymphadenopathies were found in the mesentery and para-aortic areas, with no signs of residual masses at the right frontal, temporal, intraorbital, and costal areas (Figure 5). According to the FDG PET/CT results, the patient was considered a partial complete response. Two more cycles were added, and alectinib was continued.

## Discussion

The ALK gene and protein

ALK was first reported by Morris et al. in 1994, where a structural change was found in the gene fusion of nucleophosmin-1 (NPM1) on chromosome 5q35 with an unidentified tyrosine kinase protein on chromosome 2p23 in anaplastic large cell lymphoma (ALCL) patient cell lineages [12]. The protein was then termed ALK, which is a protein normally found in the mature nervous system, small intestine, testes, colon, and prostate, but not in lymphoid cells and lungs [12]. It was not until 1997 that a study on wild-type ALK was reported, which then categorized it into the receptor tyrosine kinase (RTK) family due to its tyrosine kinase activity [13]. ALK, as an RTK, is part of the dependent receptor group. It functions in cell proliferation and survival upon ligand binding but is proapoptotic in the absence of a ligand [14]. ALK belongs to the LTK receptor subfamily of RTKs, comprising LTK and ALK [13,15]. Not found in other RTKs, ALK has two meprin/A5-protein/PTPmu (MAM; amino acids 264-427, 480-626) extracellular domains located between low-density lipoprotein receptor class A (LDL; amino acids 4530471) [13,15].

ALK gene mutation and activation

Unlike other RTKs, the activator ligand on ALK has not been established. Even though pleiotrophin and midkine (small heparin-binding growth factors) were previously reported as ALK-activating ligands, some studies had inconsistent conclusions [16]. Reshetnyak et al. and Guan et al. in 2015 introduced new ligands on RTK, ALK, and LTK, which are the AUG-α and β (FAM150) [16,17]. ALK has been proven to be activated by in vitro AUG-α expression, but not by AUG-β, which activates LTK more [16]. ALK, which is activated through phosphorylation due to homodimerization (transphosphorylation of a specific tyrosine residue in the ALK cytoplasmic domain), will activate several growth pathways important in cell survival, proliferation, angiogenesis, and apoptosis [16-18].

ALK mutations could be found in several forms, including rearrangements/fusion, amplification, and activator kinase mutation [18]. ALK fusion gene occurs in 0.5-0.8% of all cancer types, such as non-small cell lung cancer, lymphoma, renal cell carcinoma, thyroid carcinoma, pancreatic adenocarcinoma, inflammatory myofibroblastic tumor, and other malignancies [13,16]. In lymphoma, the two entities derived from ALK rearrangements are ALK^+^-ALCL and ALK^+^-LBCL [4]. Amplification is frequently found in neuroblastoma, breast carcinoma (especially triple-negative breast carcinoma), and inflammatory breast cancer. Another type of ALK alteration, which is the truncation of the ALK gene that eliminates the extracellular domain, could activate downstream pathways through ligand-independent mechanisms mainly found in neuroblastoma, melanoma, and squamous cell carcinoma of the skin [13,18].

ALK^+^-LBCL

ALK^+^-LBCL was first described by Delsol et al. in 1997, where there were around seven cases of diffuse large cell lymphoma with morphology similar to ALCL, less CD30 expression in immunophenotyping, and high expression of EMA reported [19]. According to the WHO 2022 classification, ALK^+^-LBCL is a monomorphic neoplasm of large diffuse B cells with plasmablastic immunophenotype and ALK expression due to ALK gene rearrangement. Some literature also added CD20-negative into the definition [4,8]. In our case, a 13-year-old boy was diagnosed with ALK^+^-LBCL based on histopathology and extensive immunohistochemistry.

This lymphoma type is very rare, accounting for less than 1% of adult NHL [4,20]. There is a male predominance (3.5:1) found in this disease [21,22]. The average age at diagnosis is around 38-40 years, but no age distribution has been established in children [7]. Very rarely is this disease found in children; a meta-analysis by Castillo et al. showed that only 10% of cases were in the pediatric age group, whereas Jiang et al. found only one pediatric case in 17 cases [7,23].

The typical ALK^+^-LBCL manifestation involves only the painless enlargement of lymph nodes (56% of all cases), which most commonly presents in the cervical area (73%) [7,24]. Around 30% of cases had both nodal and extranodal involvement [7]. Primary extranodal involvement could only be found in 14% of cases, notably in the upper airway, bone, and gastrointestinal tract [7]. Although less common, other varieties of sites may include the eyes, central nervous system, spleen, and liver [7,25]. B symptoms could also be found in half to two-thirds of patients [23]. In almost one-third of patients at the time of diagnosis, bone marrow involvement is typically found [4]. Blood chemistry often shows elevated LDH and beta-2 microglobulin [4,6]. This disease worsens more rapidly compared to other LBCLs, with patients typically presenting in advanced stages (60-78%) [23]. Our patient complained of fever and worsening abdominal pain. Tenderness was found in the RLQ, initially suspected of being appendicitis. Although primary gastrointestinal NHL is one of the most common extranodal sites of NHL (30-40% of cases), appendicitis presentation is extremely rare, representing only 0.015% of all LBCLs [26]. Afterward, both nodal and extranodal involvement were found, yet in uncommon sites such as the bone and intraorbital areas. Bone marrow tumor involvement was also found from aspiration.

Understanding of ALK^+^-LBCL tumorigenesis is limited due to its rarity. Studies have shown excessive signal transducer and activator of transcription 3 (STAT3) and 5 (STAT5) protein expression in ALK^+^-LBCL patients, suggesting that ALK gene fusion with partner genes such as clathrin heavy chain will activate the Janus kinase-signal transducers and activators of transcription (JAK/STAT) pathway [27]. A high expression of STAT3 (<50% of cases) in ALK will increase plasma cell differentiation and subsequently increase the XBP1 gene and reduce the PAX5 gene, thus related to its plasmablastic phenotype [28]. The XBP1 gene is an important transcription factor in B cell differentiation, but a decreased PAX5 is needed for plasma cell differentiation [28].

ALK^+^-LBCL is diagnosed through various histopathological and immunohistochemical examinations. Histopathology shows a diffused large round cell tumor with increased nuclear and cytoplasmic ratio, leading to a malignant round cell tumor diagnosis by conventional staining [29,30]. Morphologically, this lymphoma could diffusely replace the normal architecture in 97% of cases, but lymph node involvement in 1/3 of cases shows a sinusoidal growth pattern [4]. The neoplastic cells in ALK^+^-LBCL include large and monomorphic cells, with amphophilic or eosinophilic cytoplasm [4]. These cells have centrally located nuclei with prominent immunoblastic-like nucleoli or large cytoplasmic cells with eccentric nuclei [4]. Castillo et al. reported large cells in 85% of cases, with oval to round nuclei (95%), centrally located nuclei (53%), and prominent nucleoli in all cases [7]. Abundant cytoplasm could be found in almost all cases (98%), either eosinophilic (52.3%) or basophilic (48%) [7]. Reed-Sternberg-like cells were also reported in 60% of cases [7]. Scattered small lymphocytes and mature plasma cells were also found in 81% of cases as a background among the neoplastic cells [7]. A study by Pan et al. in 2017 presented tumor cells in the lymph nodes forming cohesive sheets or nests resembling non-hematolymphoid malignancies (epithelioid) [21,31]. A high mitotic index could be found in 96% of cases, while necrosis was observed in 20-50% of cases [4,7].

The differential diagnosis, according to morphology and tumor size, includes undifferentiated carcinoma (metastasis), melanoma, large cell sarcomas, and lymphoma [29,30]. Some NHL, including ALCL, diffuse large B-cell lymphoma (DLBCL), and plasmablastic lymphoma (PBL), could share the same morphology (being plasmablastic/immunoblastic) [9]. This considerable overlapping morphology emphasizes the importance of immunohistochemistry in attaining a diagnosis.

The choice of antibodies for immunohistochemistry is based on clinical and morphological settings. In this case, a lymphoid malignancy is suspected due to the high nodal involvement and disease demographics. For an undifferentiated large cell tumor, recommendations include the use of cytokeratin (CK) to exclude carcinoma, vimentin for sarcoma and other hematolymphoid tumors, CD45/leucocyte common antigen (LCA) for lymphoid tumors, and Sal-like protein 4 (SALL4) for germ cell tumors, among others (Table 1) [32-34]. Lin and Liu also recommended using CD43 to detect additional suspected lymphomas or myeloid sarcoma antibodies [32]. Furthermore, CD138, MUM1, and ALK were also added according to the pathological morphology and clinical features [8,33]. Immunohistochemistry of this patient resulted in the weak expression of LCA, negative pan-B and pan-T antigens, and positive tumor cell staining for CD10, CD138, CD38, MUM1, EMA, ALK, and Ki67 (70%). The EMA immunophenotype was positive, but MUM1 positivity helps rule out the consistently negative carcinoma diagnosis [35]. Plasma cell marker CD138 is almost always positive (98%) in ALK^+^-LBCL and PBL, but often negative in Kaposi sarcoma-associated herpesvirus/human herpesvirus 8 (KSHV/HHV8)-positive DLBCL [4,7]. CD10 was occasionally found to be positive in 46% of patients [7]. While this case shares almost the same immunophenotype (negative B marker and positive for plasma cell marker) with PBL, ALK can rule out PBL from the diagnosis [4,6]. When PBL cells are typically EMA and CD79a positive, only 21% of ALK^+^-LBCL are CD79a positive [7]. In addition, PBL often occurs in immunodeficiency contexts such as HIV infection (31-62%), history of an immunosuppressive agent, and Epstein-Barr virus (EBV) infection, mostly affecting the nasal/oral cavity (>52% cases) [4,5].

Although previous studies reported that all cases were negative for EBV infection, a recent study from Wu et al. found a case of ALK^+^-LBCL with an EBV infection proven through in situ hybridization (ISH) for EBV-encoded ribonucleic acid (EBER) [36]. This rare case can lead to a misdiagnosis of EBV+DLBCL [36]. Loghavi et al. showed a high Ki-67 proliferation index in PBL with a median value of 90%, while it ranges from 50-90% in ALK^+^-LBCL [8,37]. ALK^+^-ALCL was excluded due to CD30 negativity and plasma cell marker positivity, even though CD30 can be positive in 14% of ALK^+^-LBCLs [7].

Neoplastic cells in ALK^+^-LBCL will show positivity in plasma cell markers, such as CD138, VS38c, XBP1, BLIMP1, and MUM1. CD20 is found positive in 10-25% of cases, with ALK-positive staining helping differentiate it from other CD20-negative LBCL types, such as PBL, primary effusion lymphoma, and HHV-positive DLBCL [4,8]. Immunophenotyping with EMA, OCT-2, BOB.1, cytoplasmic immunoglobulin (IgA > IgG; lambda > kappa), CD45RB, STAT3, and MYC could also be positive [4].

The ALK pattern in immunohistochemistry staining could be related to the partner gene type in ALK gene translocation. Wang et al. and Jiang et al. reported that the partner gene most commonly found is CLTC (66-75%), followed by NPM1(6-10%) [23,27]. CLTC-ALK gene fusion will result in positive ALK staining with a granular pattern in the cytoplasm, while NPM1-ALK will have positivity in the nuclei and cytoplasm [23,27]. Positive CD15, weak expression of PAX5, and negative CD45 staining in Reed-Sternberg cells (classical Hodgkin lymphoma) help differentiate the Reed-Sternberg-like cells in these cases [38].

Currently, there is no standard therapy for ALK^+^-LBCL. Based on the WHO 2022 classification, ALK^+^-LBCL therapy is limited to systemic chemotherapy using the CHOP regimen [4]. However, the NCCN 2024 guidelines recommended CHOP treatment in the earlier stages, followed by radiotherapy, and advanced stages treated with CHOP alone, with consideration of radiotherapy in the bulky areas of the disease [8]. Other chemotherapy regimens include dose-adjusted-etoposide phosphate, prednisone, vincristine sulfate (oncovin), cyclophosphamide, and doxorubicin hydrochloride (hydroxydaunorubisin) (DA-EPOCH), cyclophosphamide, doxorubicin, etoposide, vincristine, and prednisone (CHOEP), mini-CHOP, cyclophosphamide, vincristine, sulfate, doxorubicin hydrochloride/adriamycin, methotrexate, cytarabine, and dexamethasone (hyperCVAD), and cyclophosphamide, vincristine, doxorubicin-high dose methotrexate/ifosfamide, etoposide, and high dose cytarabine (CODOX-M/IVAC) that could be used in this condition. Several studies have shown that the CHOP regimen has a poorer clinical outcome than other regimens, but it is considered for cases in the early stages combined with other treatment modalities (e.g., radiotherapy for localized lesions) [8,23]. In relapsed/refractory cases, second-line platinum-based chemotherapy (without rituximab), continued with high-dose chemotherapy with autologous stem cell rescue (HDT/ASCR), is recommended for transplant-eligible patients. The newest generation of ALK inhibitors, such as alectinib and lorlantinib, revealed a better outcome compared to previous generations like crizotinib [8,39]. A retrospective study by Pan et al. showed 134 cases of ALK^+^-LBCL treated with chemotherapy combined with local radiotherapy, and some subjects receiving hematopoietic stem cells had a five-year overall survival (OS) of only around 34% with a median survival time of 1.83 years [21]. Wang et al. showed promising results, as three from seven patients achieved a complete response after undergoing the DA-ECHOP regimen followed by autologous stem cell transplantation (ASCT) [27]. A single case from Sandoval-Sus et al. reported a complete response from a relapse/refractory case with single-agent activity of checkpoint inhibitors (nivolumab), and the patient remained disease-free after 14 months of treatment [11]. 

The use of ALK inhibitors is still inconclusive. ALK inhibitors are anticancer drugs that act on the ALK kinase domain by preventing phosphorylation [40]. There are three known generations of ALK inhibitors: first-generation (crizotinib), second-generation (ceritinib, alectinib, brigatinib, and ensartinib), and third-generation (lorlatinib) [41]. There has been no large study on the role of crizotinib in ALK^+^-LBCL. A case study by Wass et al. reported its use on a short-term response after three weeks, with increased LDH levels and new nodal involvement [42]. Yet the patient died within one month due to massive progression of the disease [42]. Xia et al. also reported remission and short-term clinical improvement with crizotinib, but resistance developed within less than one month of treatment [43].

The second-generation ALK inhibitor alectinib is considered to have better efficacy than crizotinib [44]. In a phase II clinical trial by Fukano et al., the drug demonstrated a one-year progression-free survival (PFS) and OS rate of 58.3% (90% CI: 28.6-79.3) and 70.0% (90% CI: 39.6-87.2), respectively [44]. Safety factors received special attention, as visual disturbances found in 23% of patients receiving first-generation ALK inhibitors were not reported with alectinib use [44]. Zhang et al. reported pediatric patients with a combination of modified LMB89 group C regimen and alectinib, followed by allogeneic hematopoietic stem cell transplantation (allo-HSCT) after second episodes of relapse [24]. They remained in a complete response state after 16 months of undergoing allo-HSCT [24]. This study also recommended that allo-HSCT be performed as soon as remission is achieved [24]. Soumerai et al. reported four relapsed/refractory cases, with one achieving a partial response and three patients achieving a complete response after using alectinib 600 mg twice daily [39]. One patient underwent allo-HSCT and experienced progression of the disease, then achieved a complete response after switching to lorlatinib 100 mg per day [39]. A recent report described combination therapy with alectinib plus vincristine, rituximab, cyclophosphamide, and doxorubicin (VRCD), in combination with brentuximab vedotin, based on CD30 expression in approximately 25% of tumor cells, achieving a complete response after two treatment cycles [45]. However, this regimen is not applicable to our case because of the absence of CD30 expression.

In our case, NSE and LDH levels are increased. LDH is commonly elevated in lymphoma and ALK^+^-LBCL [4]. NSE levels are typically elevated in neurogenic and neuroendocrine origins, but recent studies showed NSE levels in DLBCL lines were higher than normal B-cells (control) [46,47]. A study also proposed that the role of NSE in DLBCL is not by inducing direct biological changes, but through interactions with the tumor microenvironment and regulating tumor-associated macrophages polarization [48]. The role of NSE specifically in ALK^+^-LBCL has not been reported before; however, NSE levels were found to be increasing along with the progression of disease in our case. Further research is needed to identify the role and its potential as a prognostic factor or therapy.

The prognosis in ALK^+^-LBCL is poor due to both adult and pediatric cases having a lack of effective standard therapy. Pan et al. reported a retrospective study of 134 cases (26 from institutional cases, 108 from the literature) that showed a five-year OS of 34% with a median survival of 1.83 years [21]. They also reported how the clinical stage and age of onset have a significant relationship: the five-year OS for the earlier stage was 66%, and for the advanced stage was 8% [21]. Granular cytoplasmic staining of ALK is the most frequent pattern in ALK^+^-LBCL with CLTC as a fusion gene partner [22]. A study that looked into 112 cases found that a group with granular cytoplasmic patterns was significantly associated with a better outcome, even after age and clinical stages were adjusted [49].

## Conclusions

ALK^+^-LBCL is an extremely rare entity in both adult and pediatric populations. Most reported cases present at advanced stages with combined nodal and extranodal involvement. Diagnosis relies on histopathological evaluation supported by extensive immunohistochemical analysis, as the disease may mimic both non-hematolymphoid neoplasms and other lymphomas. The prognosis remains poor, largely due to the absence of an established standard therapeutic approach for either adults or children. In the present case, ALK^+^-LBCL occurred in a pediatric patient and achieved a sustained partial response for more than one year following combination therapy with CHOP chemotherapy and alectinib, with acceptable tolerability. Further studies involving larger patient cohorts are warranted to clarify the clinical significance of neuron-specific enolase levels in ALK^+^-LBCL and to evaluate its potential role as a prognostic biomarker or therapeutic target.
